# Dynamic Modulation of Thymidylate Synthase Gene Expression and Fluorouracil Sensitivity in Human Colorectal Cancer Cells

**DOI:** 10.1371/journal.pone.0123076

**Published:** 2015-04-16

**Authors:** Kentaro Wakasa, Rumi Kawabata, Seiki Nakao, Hiroyoshi Hattori, Kenichi Taguchi, Junji Uchida, Takeharu Yamanaka, Yoshihiko Maehara, Masakazu Fukushima, Shinya Oda

**Affiliations:** 1 Clinical Research Institute, National Kyushu Cancer Center, Fukuoka, Japan; 2 Tokushima Research Center, Taiho Pharmaceutical Co., Ltd., Tokushima, Japan; 3 Clinical Research Center, Nagoya Medical Center, Nagoya, Japan; 4 Department of Biostatistics, Yokohama City University, Yokohama, Japan; 5 Department of Surgery and Science, Graduate School of Medical Sciences, Kyushu University, Fukuoka, Japan; Baylor University Medical Center, UNITED STATES

## Abstract

Biomarkers have revolutionized cancer chemotherapy. However, many biomarker candidates are still in debate. In addition to clinical studies, *a priori* experimental approaches are needed. Thymidylate synthase (TS) expression is a long-standing candidate as a biomarker for 5-fluorouracil (5-FU) treatment of cancer patients. Using the Tet-OFF system and a human colorectal cancer cell line, DLD-1, we first constructed an *in vitro* system in which TS expression is dynamically controllable. Quantitative assays have elucidated that TS expression in the transformant was widely modulated, and that the dynamic range covered 15-fold of the basal level. 5-FU sensitivity of the transformant cells significantly increased in response to downregulated TS expression, although being not examined in the full dynamic range because of the doxycycline toxicity. Intriguingly, our *in vitro* data suggest that there is a linear relationship between TS expression and the 5-FU sensitivity in cells. Data obtained in a mouse model using transformant xenografts were highly parallel to those obtained *in vitro*. Thus, our *in vitro* and *in vivo* observations suggest that TS expression is a determinant of 5-FU sensitivity in cells, at least in this specific genetic background, and, therefore, support the possibility of TS expression as a biomarker for 5-FU-based cancer chemotherapy.

## Introduction

Biomarkers predicting patient outcomes now play an essential role in various medical fields, particularly in target-based cancer therapies. Biomarkers are regarded as reflecting the structural and functional states of target molecules and those functioning up/downstream of them. Currently, according to the status of biomarkers, patients are stratified and treated in several neoplastic diseases. However, the accuracy of prediction is not yet satisfactory in general. One reason is that, in many of the target molecules, their roles in drug sensitization are not well clarified, and that the physiological functions are also not well understood in some molecules. Secondly, the structures and functions of genes encoding the target molecules change in tumor cells and, consequently, vary in the patient populations. Thirdly, we point out that biomarkers have not thus far been approached quantitatively. Quantitative analyses and theorization are essential for a more precise prediction of patient outcomes. More quantitative assay techniques are required. These problems are identified as a major obstacle to the development of biomarker-driven strategies for personalized treatment of cancer patients. Of these three problems, the first (*i*.*e*. which biomarkers are relevant) is the most critical.

Biomarkers are thought to play an important role not only in target-based therapies but also in treatment using classical cytotoxic anticancer agents. Several molecular events in cancer have long been regarded as biomarker candidates for classical anticancer drugs. However, they all are not conclusive and still in debate, and, consequently, none of them has been introduced into clinical practice. One typical example is TS expression in tumor cells. TS is a key enzyme functioning in one of the nucleotide biosynthesis pathways and physiologically converts deoxyuridine monophosphate (dUMP) to deoxythymidine monophosphate (dTMP) by the reductive methylation using 5, 10-methylene tetrahydrofolate (CH(2)THF) [1]. 5-FU is one of the most widely and frequently used antineoplastic agents classified as antimetabolites. Once incorporated into cells, 5-FU is metabolized into 5-fluorodeoxyuridine monophosphate (FdUMP). This metabolite, FdUMP, forms a stable enzyme-substrate intermediate with TS and CH(2)THF, which strongly inhibits the thymine biosynthesis pathway and, consequently, causes a depletion of dTTP in the nucleotide pool [2,3], possibly leading to an inhibition of DNA replication. Intriguingly, TS is variably expressed in human tumors [4–6]. This fact implies that response to 5-FU treatment may vary widely depending on the expression level of TS in each tumor. In order to explore the possibility of TS as a biomarker for 5-FU-based chemotherapy, numerous clinical studies have been done using tumor tissue samples. However, the results reported in the literature were diverse (see Discussion). Currently, TS is not regarded as a promising biomarker candidate [7]. We address this problem in the present study. Instead of clinical (*i*.*e*. *a posteriori*) approaches, we adopted an *in vitro* experimental (*i*.*e*. *a priori*) approach in this study. Using a transgene (*i*.*e*. an artificial gene introduced into cultured cells), TS expression was variably modulated in a single genetic background. Although TS transgene has previously been addressed in a pioneer study by Johnston and colleagues [8], we first constructed an *in vitro* system in which TS expression is dynamically controllable. We examined 5-FU sensitivity of human colorectal cancer cells when TS expression is widely modulated. Obtained data clearly suggest that 5-FU sensitivity changes according to the expression level of TS in cells, and that, in other words, TS expression is a determinant of 5-FU sensitivity, at least in the selected genetic background. Here, we report an *a priori* demonstration of the role of TS expression in cellular sensitivity to 5-FU. Our results support the possibility of TS expression as a biomarker for 5-FU-based cancer chemotherapy.

## Materials and Methods

### Chemicals

Hygromycin B (HygB), G418 and doxycycline (Dox) were purchased from Clontech Laboratories, Inc. (Mountain View, CA, USA). Dox used for animal studies was purchased from MP Biomedicals, LLC. (Santa Ana, CA, USA). [6-^3^H]-5-Fluoro-2’-deoxyuridine 5’-monophosphate ([6-^3^H] FdUMP) and [5-^3^H]-2’-deoxyuridine 5’-monophosphate ([5-^3^H] dUMP) were obtained from Moravek Biochemicals, Inc. (Brea, CA, USA). The oral 5-FU prodrug composed of tegafur, 5-chloro-2,4-dihydroxypyridine (CDHP) and potassium oxonate, S-1 [9], was produced in Taiho Pharmaceutical Co., Ltd. (Tokyo, Japan). All other chemicals were purchased from Sigma Chemical Co. (St. Louis, MO, USA) unless indicated otherwise.

### Plasmid construction

**Fig 1:**
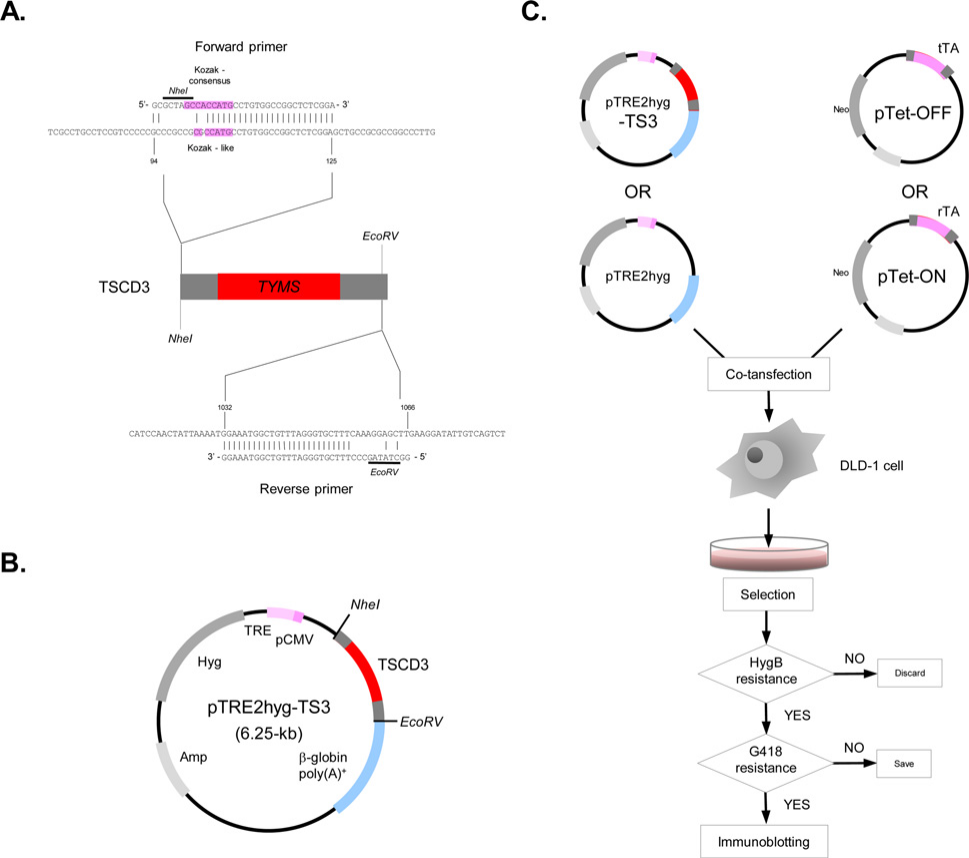
Transformation strategy. **A.** The original Kozak-like motif in the human *TYMS* cDNA was modified to the Kozak consensus sequence using partially complementary PCR primers (see Materials and methods). **B.** The Kozak-modified *TYMS* cDNA, TSCD3, was amplified by PCR and subcloned into the cDNA expression vector, pTRE2hyg. **C.** pTRE2hyg-TS3 or an empty vector was co-transfected with pTet-ON/OFF vectors into a human colorectal cancer cell line, DLD-1. After serial selections, clones resistant to both HygB and G418 were isolated. TS expression in these clones was then examined by immunoblotting.

The plasmid carrying 1.6 kilobase pairs (kb) *TYMS* cDNA fragment, pcHTS1, has previously reported [10]. From this plasmid, a 1.0 kb fragment was amplified by polymerase chain reaction (PCR), using mismatch primers that alter the original Kozak-like motif in the 5’ untranslated region to the Kozak consensus sequence [11] and generate new restriction sites, *NheI* and *EcoRV* (Fig 1A). This *NheI*-*EcoRV* fragment including the modified *TYMS* cDNA, TSCD3, was subcloned into the cDNA expression vector in the Tet system, pTRE2hyg, which is commercially provided by Clontech Laboratories Inc. (Fig 1B). The constructed vector was designated as pTRE2hyg-TS3. The vectors expressing the Tet transactivators, pTet-ON and pTet-OFF, were also obtained from Clontech Laboratories Inc.

### Cell culture and transformation

Human colorectal carcinoma cell line, DLD-1, was obtained from American Type Cell Culture Collection (Manassas, VA, USA). Cells were cultured in RPMI1640 media supplemented with 10% fetal bovine serum (FBS). The media was purchased from Life Technologies (Carlsbad, CA, USA). Using Lipofectamine 2000 (Life Technologies), pTRE2hyg-TS3 and pTet-ON/OFF vectors were co-transfected into DLD-1 cells, according to the manufacturer’s instructions. After transfection, cells were replated at the previously determined density. At 24h after replating, 350 μg/ml HygB was added to the media. HygB-resistant colonies were recovered using cloning cylinders and plated onto 3.5cm dishes. These small-scale cultures were then divided into two: one was stored in liquid nitrogen and the other forwarded to the secondary selection using G418. 350 μg/ml G418 was similarly added to the media and G418-resistant clones were recovered. All the transformants were maintained in RPMI1640 media supplemented with 10% FBS, 50 μg/ml HygB and 50 μg/ml G418.

### Immunoblotting

**Fig 2:**
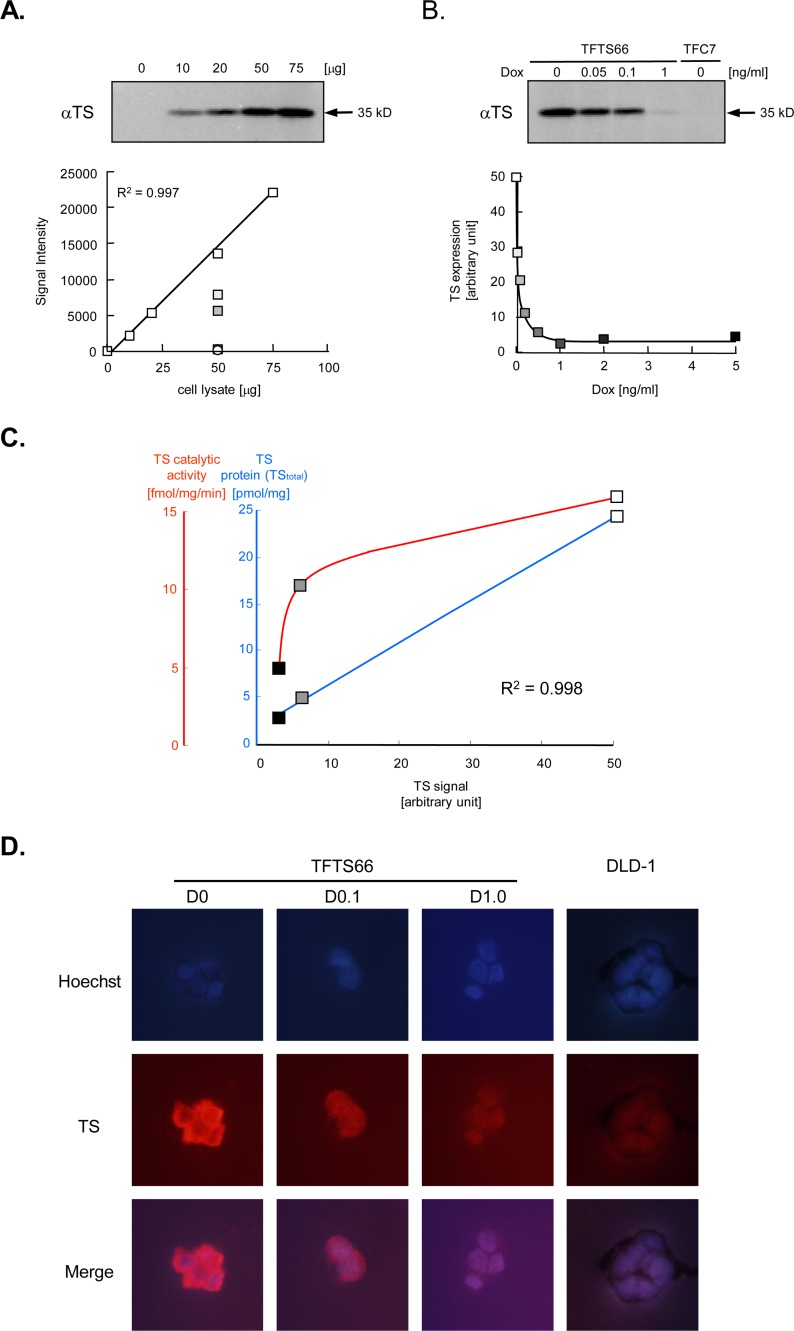
Dox-dependent TS expression in TFTS66 cells. **A.** TS antigens in cell lysates of TFTS66 transformant were detected by immunoblotting using anti-human TS mouse monoclonal antibody. The standard cell lysates (0 ng/ml Dox) were titrated, and a standard curve for detection was obtained from the signal intensity on the digitized image (upper panel). Using the highly linear detection characteristics (p = 0.997), TS expression levels were quantified: rectangle, TFTS66; circle, the control transformant, TFC7. **B.** TS expression in TFTS66 cells exposed to various concentrations of Dox was assessed by immunoblotting and similarly quantified. The symbols are shaded according to the Dox concentrations. Some of the data points are also shown in Fig 2A. **C.** The quantity of TS protein (TS_total_, see Materials and methods) and its catalytic activity were enzymatically assayed in lysates prepared from TFTS66 cells exposed to 0, 0.5 and 1.0 ng/ml Dox. The results are plotted as a function of the TS expression level determined by immunoblotting: open rectangle, Dox0; shaded rectangle, Dox0.5; closed rectangle, Dox1.0. **D.** TS expression in TFTS66 cells was observed using fluorescent immunocytochemistry. Cells grown on chamber slides were fixed and reacted with TS-specific antibody. Cellular distribution of TS antigens was visualized by red fluorescent signals. Cells were also counterstained with Hoechst 33342. Results obtained in TFTS66 (0, 0.1 and 1.0 ng/ml Dox) and its parental line, DLD-1, are shown (magnification X100).

Cells were washed twice with 0.02% EDTA in phosphate-buffered saline (PBS), pelleted and kept at –80°C until use. Cell pellets were lysed in 2X Laemmli's sodium dodecyl sulphate (SDS) sample buffer [12], sonicated and then cooled on ice. After centrifugation at14,000 x *g* for 30 min at 4°C, supernatants were collected. Lysates corresponding to 50 μg were subjected to SDS-polyacrylamide gel electrophoresis (PAGE) and separated proteins were electrotransferred onto nitrocellulose membranes, BA83 (GE Healthcare Bio-Science Corp., Piscataway, NJ, USA), using Transblot SD (Bio-Rad Laboratories, Hercules, CA, USA). After blocking with 1X TBS (10 mM Tris-HCl; pH 7.4, 0.9% NaCl) solution including 5% bovine serum albumin (BSA) and 0.05% Tween 20 at 52°C for 1 h, the membranes were reacted with appropriately diluted primary antibody solutions at 4°C overnight. The membranes were then incubated with a horseradish peroxidase-conjugated protein A (GE Healthcare Bio-Science Corp.) or anti-rabbit immunoglobulin G secondary antibody (Santa Cruz Biotechnology Inc., Santa Cruz, CA, USA), and the resulting bands were visualized using the ECL Advance kit (GE Healthcare Bio-Science Corp.) and scanned by a CCD camera in the Chemi Doc system (Bio-Rad Laboratories). The signal intensity of each band was quantified on digitized images using Molecular Analyst software (Bio-Rad Laboratories). In each assay, in addition to experiments, 75, 50, 20, 10 and 0 μg of standard cell lysates were run on a same membrane and probed as a standard curve for detection. When the detection characteristics obtained from cell lysate titrations are highly linear, antigens are detected quantitatively. Using linear detection characteristics, the expression level corresponding to each experiment can be estimated by interpolation and relatively quantified (see Fig 2A). Mouse monoclonal antibody raised against recombinant human TS has previously been described elsewhere [13] and was obtained from Immuno-Biological Laboratories Co., Ltd. (Gunma, Japan).

### RNA extraction and expression microarrays

Cells were washed twice with PBS, pelleted and kept in liquid nitrogen until use. Total RNA was extracted from the cell pellets using ISOGEN (Nippon Gene, Toyama, Japan), according to the manufacturer’s instructions. The quality of extracted RNA was confirmed using the Agilent 2100 Bioanalyzer (Agilent Technologies Inc., Santa Clara, CA, USA) and Agilent RNA Nano Chips (Agilent Technologies Inc.). In all RNA samples, 28S, 18S, 5.8S and 5S rRNA and tRNA were clearly observed, and RIN values were above 9.0. In each microarray analysis, the GeneChip Human Genome U133 Plus 2.0 array (Affimetrix, Santa Clara, CA, USA) was used, and 250 ng of total RNA was subjected to the system.

### Quantity and activity of TS

Enzymatic quantification of TS protein using radiolabelled FdUMP, *i*.*e*. TS binding assay, was originally described by Spears P *et al*. [14]. The TS quantity was assessed, largely according to their methods. Briefly, cells or tumor tissues were homogenized and sonicated. After centrifugation at 105,000 x *g* for 60 min, supernatants were recovered, and the protein concentration was determined by the method of Bradford [15]. The supernatants were then reacted with [6-^3^H] FdUMP in the presence of CH(2)THF at 30°C for 20 min. The radioactivity in the acid-insoluble fraction was determined by liquid scintillation counting (TS_free_). In order for TS proteins bound to FdUMP to dissociate, the supernatants were pre-incubated in a buffer containing NH_4_HCO_3_ at 25°C for 3 h, and reacted with [6-^3^H] FdUMP. The radioactivity was similarly determined (TS_total_).

We have previously established a TS catalytic activity assay based on Dunlap RB *et al*.’s method [16]. Briefly, crude cell extracts were reacted with CH(2)THF and [5-^3^H] dUMP in potassium phosphate buffer (pH6.8) containing sodium fluoride. After an incubation period of 10 min at 34°C under nitrogen, the reaction was stopped by the addition of perchloric acid. Activated charcoal was added to the mixture and removed by centrifugation. The radioactivity of the supernatants was determined by liquid scintillation counting.

### Flowcytometry

Cells were harvested by trypsinization and resuspended in 1.0 ml of the buffer containing 3.4 mM sodium citrate, 10 mM NaCl, 0.1% Nonidet (N)P-40 and 50 mg/ml of propidium iodide. After incubation for 2–3 h at 4°C, samples were subjected to BD FACScan flowcytometer (Becton Dickinson and Company, Franklin Lakes, NJ, USA). For each sample, 10 000 cells were analyzed, and the results were processed using analytical software, CellQuest (Becton Dickinson and Company).

### Colony formation assay

Cells were seeded onto 10 cm dishes at an initial density of 5 x 10^4^ cells per dish. After incubation for 72 h, cells in an exponential growth phase were treated with 5-FU for 72 h, by replacing media with those containing various concentrations of 5-FU and then with those without the agent. After 10 days, formed colonies were fixed in 3% buffered formaldehyde, stained with 0.1% methylene blue and counted. The experiments were done in triplicate for each point, and means and standard errors were calculated.

### Immunocytochemistry

Cells were seeded onto Nunc Lab-Tek Chamber Slide (Thermo Fisher Scientific, Waltham, MA, USA) at an initial density of 5 x 10^3^ cells per slide and grown for 3 days. After fixing with 2% paraformaldehyde in PBS, cells were permiabilized with 0.1% Triton X-100 in PBS at room temperature (RT) for 20 min. Blocking was performed by treating slides with PBS containing 10% goat serum, 0.5% BSA, 0.15% glycine for 20 min. Fixed cells were next reacted with 2 μg/ml anti-human TS mouse monoclonal antibody TS106 (Abcam plc, Cambridge, United Kingdom) at RT for 1h, followed by washing with PBS supplemented with 0.5% BSA and 0.15% glycine (PBG), and then incubated with 1:100 diluted secondary antibody, Qdot 655 goat F(ab’)2 anti-mouse IgG conjugate (H+L) (Life Technologies), at RT for 1 h. After washing, cells were stained with 0.01% Hoechst 33342 (Life Technologies) and mounted using Fluorescent Mounting Medium (Dako Denmark A/S, Glostrup, Denmark). TS expression in cells was observed under fluorescence microscopy.

### Immunohistochemistry

Tumor xenograft tissues were fixed in 10% buffered formalin for 24 h and embedded in paraffin. Formalin-fixed, paraffin-embedded (FFPE) tissue specimens were sectioned at 3μm-thickness and mounted onto silanized glass slides. The slides were heated in citrate buffer (pH6.0) using a pressure cooker for 30 sec at 125°C and then for 10 sec at 90°C, followed by cooling at RT for 30 min. Endogenous peroxidase activity was blocked with 3% hydrogen peroxide for 5 min. Sections were then reacted with 1:100 diluted anti-human TS mouse monoclonal antibody (Immuno-Biological Laboratories Co., Ltd.) for 60 min, followed by washing. After incubation with anti-mouse peroxidase-labelled IgG (Dako Denmark A/S) for 30 min. the antigen was visualized using diaminobenzidine tetrahydrochloride (DAB) (Dako Denmark A/S). Finally, sections were counterstained with Mayer’s hematoxylin (Dako Denmark A/S). TS expression in tumor tissues was observed under light microscopy and photographed using an automated digital slide scanner, NanoZoomer 2.0-HT (Hamamatsu Photonics K.K., Hamamatsu, Japan) Digitized images were viewed using NanoZoomer Digital Pathology (NDP) software (Hamamatsu Photonics K.K).

### Animal studies

All animal procedures were performed in accordance with the Guide for The Care and Use of Laboratory Animals (National Research Council 1996) and approved by the Animal Care Committee, Tokushima Research Center, Taiho Pharmaceutical Co., Ltd. (Tokushima, Japan). Five-week old male nude mice (CAnN.Cg-*Foxn1* ^nu^/CrlCrlj) were obtained from Charles River Laboratories Japan, Inc. (Yokohama, Japan). Animals were maintained under controlled lighting (12 h light/dark cycle), temperature (23+/–3°C) and humidity (50 +/–20% RH). Food and water were provided *ad libitum*. At 7 weeks of age, animals were randomly assigned to six groups (see Table 1). TFTS66 cells (1.15 x 10^7^ cells/mice) were subcutaneously inoculated into the back of each animal. Dox was dissolved in saline, filtrated and administered intraperitoneally to the animals for 14 consecutive days. S-1 was prepared in 0.5% hydroxypropyl methylcellulose (HPMC) solution and orally administered to the animals. Body weight was measured twice a week throughout the experimental period. At day 8, the control and Dox-treated animals (*n* = 4 in each group) were sacrificed, and TFTS66 cell xenografts were recovered and subjected to TS binding assay and immunohistochemistry. At day 15, all the tumor xenograft tissues were resected, and their weight was measured. Resected tissues were divided into two parts: one being immediately frozen in liquid nitrogen for TS binding assay and the other fixed in formalin solution for immunohistochemistry. All animals used in this study were euthanized by exsanguination under isoflurane anesthesia or by inhalation of carbon dioxide gas.

**Table 1:** Anti-tumor effects of 5-FU in mice carrying TFTS66 cell xenografts.

Group	Dox [mg/kg/day] *i*.*p*.	Tegafur^a^ [mg/kg/day] *p*.*o*.	No. of animals	No. of deaths	Tumor weight (mean ± s.e.) [g]	p value^b^	p value^c^	TGI^d^ [%]	BWC^e^ (mean ± s.e.) [%]
A	–	–	8	0	1.06 ± 0.19	–		–	-1.03 ± 5.00
B	60	–	8	0	1.05 ± 0.18	1.00		1.0	-7.32 ± 3.85
C	–	8.3	8	0	0.86 ± 0.13	0.032		18.6	-2.44 ± 3.85
D	–	10	8	0	0.78 ± 0.09	0.001		26.2	-0.18 ± 4.59
E	60	8.3	8	0	0.69 ± 0.09	<0.001	0.008	35.2	-9.79 ± 6.31
F	60	10	8	3	0.72 ± 0.12	0.001	0.28	32.6	-7.96 ± 4.62

^a^ Tegafur, an oral prodrug of 5-FU, was administered as a combined formulation with CDHP and oxonate. 5-FU doses are expressed as those of tegafur.

^b^ Difference to the control, group A, was examined by Dunnett's test.

^c^ Difference between two 5-FU-administered groups (group E vs. C and F vs. D) was examined by Student's t-test.

^d^ Tumor growth inhibition (TGI) was calculated according to the following formula: TGI [%] = [(mean tumor weight (TW) of the control group)—(mean TW of the treated group)] / (mean TW of the control group) × 100.

^e^ Body weight change (BWC) on day 15 were calculated according to the following formula: BWC [%] = [(BW on day 15)—(BW on day 0)] / (BW on day 0) × 100.

## Results

### Establishment of a human colorectal cancer cell line expressing *TYMS* transgene variably

The expression vector carrying the Kozak-modified *TYMS* cDNA, pTRE2hyg-TS3 (Fig 1B), and pTet-ON/OFF vectors were co-transfected into a human colorectal cancer cell line, DLD-1 (Fig 1C). After serial selections with HygB and G418, we have obtained four and six resistant clones in the Tet-ON and Tet-OFF arms, respectively. We observed TS expression in these ten clones using immunoblotting. However, TS expression was not well controlled in all the four Tet-ON clones and in three Tet-OFF clones (data not shown). Thus, a Tet-OFF clone was isolated as a stable transformant that expresses human TS in a Dox-dependent manner, and designated as ‘TFTS66’. The growth rate of TFTS66 cells did not change in the range of Dox concentrations between 0 and 1.0 ng/ml (data not shown). A control transformant carrying an empty vector and pTet-OFF was similarly established and designated as TFC7.

Quantitative immunoblotting analyses are possible using cell lysate titrations (see Materials and methods). Various amounts of lysates prepared from TFTS66 cells grown in the media without Dox were probed using an antibody against TS, and the obtained detection characteristics were highly linear (R^2^ = 0.997), which indicates that TS antigens were detected quantitatively in this signal range (Fig 2A). We next probed the lysates derived from TFTS66 cells exposed to various concentrations of Dox. Intriguingly, as the Dox concentration increased, the levels of TS expression were accordingly lowered and, at the concentrations above 1 ng/ml, reached to the same level as the parental cell line, DLD-1, and the control transformant carrying an empty vector, TFC7 (Fig 2B). The ‘hyperbola-like’ dynamics of suppression had a wide range. TS expression level in the steady state (Dox0) was approximately 15-fold higher than that in cells treated with 1.0 ng/ml Dox (Dox1). It was therefore concluded that TS expression in TFTS66 cells was widely modulated depending on the Dox concentrations in the media. This has been further confirmed by the two additional methods of quantification. TS proteins are also quantifiable using radiolabelled FdUMP that irreversibly binds to this enzyme and forms a stable enzyme-substrate intermediate with folate. This TS binding assay has been widely used and is now one of the established methods of TS quantification [14]. Using this method, we quantified TS proteins in the lysates prepared from TFTS66 cells at 0 (Dox0), 0.5 (Dox0.5) and 1.0 ng/ml Dox (Dox1). Similarly to the results obtained by immunoblotting, TS proteins decreased as the Dox concentration increased. Surprisingly, TS quantities determined by TS binding assay and the results of the immunoblotting analyses were highly parallel (p = 0.998) (Fig 2C), which suggests that TS proteins are quantitatively assessed in this system. In the TS binding assays, the level of TS proteins at Dox0 was approximately ten-fold higher than that at Dox1. The catalytic activity of TS enzyme was also assayed. The TS activity can be assessed by quantifying the radioactivity released by the reductive methylation of radiolabelled dUTP [16]. The TS activity per unit protein was also high at Dox0 and low at Dox1, and, consequently, parallel to the TS quantity, although the relationship between them was not linear (Fig 2C). Thus, it has been demonstrated that TS expression in this system is dynamically controllable by varying the Dox concentration in the culture. The dynamic modulation of TS expression in TFTS66 cells was also visually confirmed using immunocytochemistry (Fig 2D).

**Fig 3:**
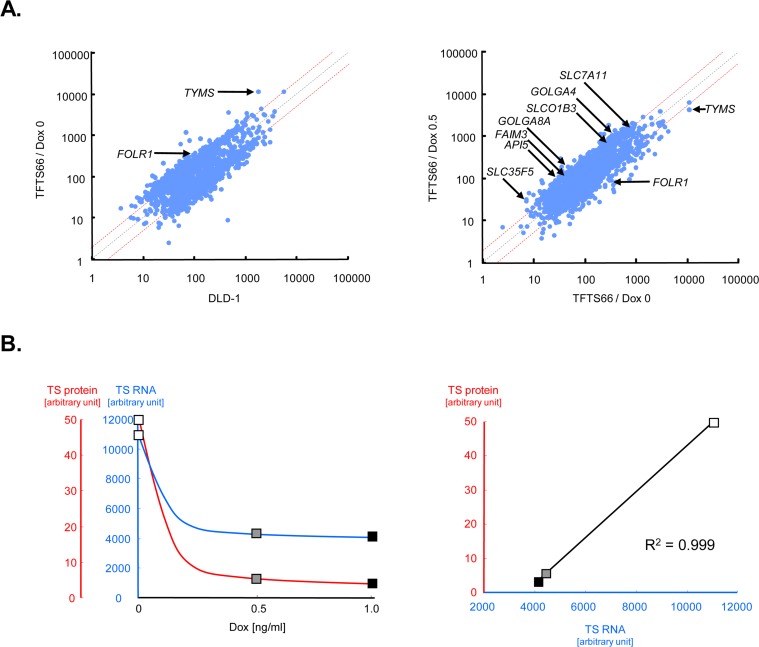
Dox effects on gene expression in TFTS66 cells. **A.** Microarray data. The expression profiles were compared between the steady state (Dox0) of TFTS66 versus the parental line, DLD-1 (left panel) and between Dox0.5 versus Dox0 in TFTS66 (right panel). Data are shown as scatter plots, and those corresponding to genes of particular interest are indicated by arrows. Red dashed lines represent the log2 fold change. **B.** The absolute values of the *TYMS* RNA level were extracted from the microarray data and are plotted against the Dox concentration, in parallel with the TS protein level determined by immunoblotting (left panel). The TS protein levels are then plotted as a function of the RNA level (right panel): open rectangle, Dox0; shaded rectangle, Dox0.5; closed rectangle, Dox1.0.

Before addressing the 5-FU sensitivity of TFTS66 cells, we examined alterations in gene expression caused by Dox exposure in this transformant. In order to observe genome-wide alterations, we adopted an expression microarray approach. The comparison between TFTS66 cells at Dox0 and its parental line, DLD-1 (Fig 3A) and that between TFTS66 cells at Dox0.5 and Dox0 (Fig 3B) were displayed in scatter plots. The level of RNA complimentary to the *TYMS* cDNA sequence was very high in TFTS66 cells at Dox0 (Fig 3A, left), but strongly suppressed in the Dox0.5 state (Fig 3A, right), confirming the above results. The TS RNA level and the TS protein quantity determined by immunoblotting were highly parallel (Fig 2B) and, intriguingly, there was a completely linear relationship between them (p = 0.999) (Fig 3B, right). Among the genes functioning in the nucleotide metabolisms, only the folate receptor 1 gene, *FOLR1*, was found to be significantly upregulated in TFTS66 cells. In parallel with the TS RNA, *FOLR1* expression was markedly downregulated in cells at Dox0.5 (Fig 3A, right). Conversely, we found that Dox exposure induces several classes of genes of particular interest. They include ones implicated in functions related to cellular transport or apoptosis. Several representative genes, the expression levels of which were more than two-fold higher in cells at Dox0.5 compared to the Dox0 state, are listed in S1 Table.

### 5-FU sensitivity of TFTS66 cells *in vitro*

**Fig 4:**
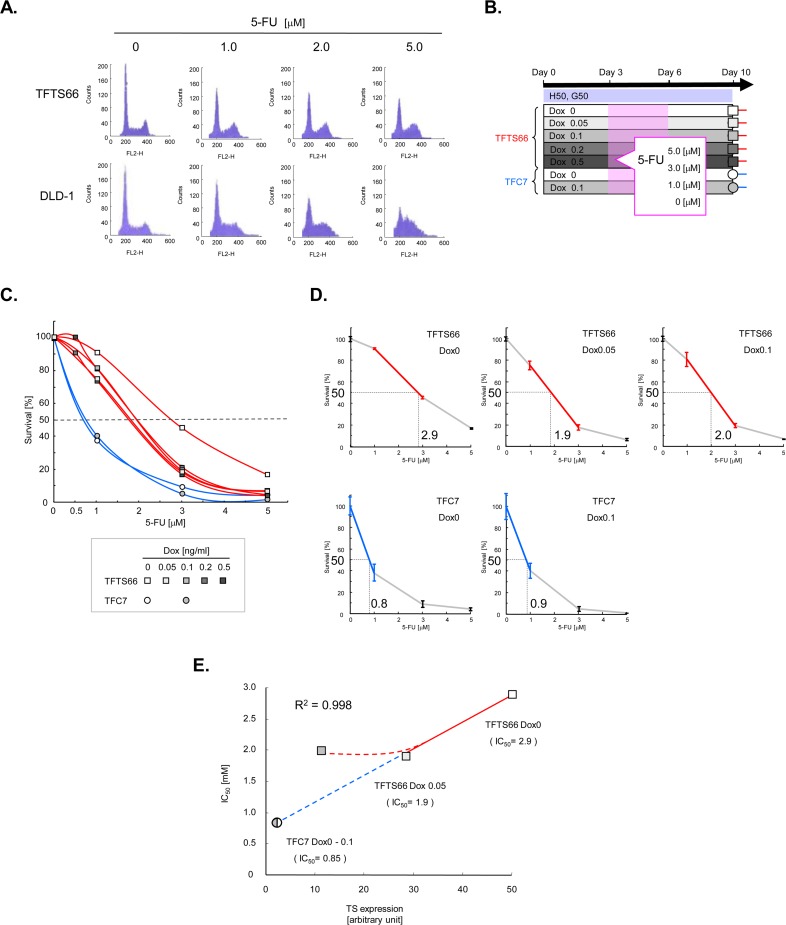
5-FU sensitivity of TFTS66 cells *in vitro*. **A.** Effects of 5-FU on the cell cycle of TFTS66 and parental DLD-1 cells. Exponentially growing cells were treated with 5-FU concentrations indicated and subjected to flowcytometry. Fluorescence histograms are shown. **B.** The design of the *in vitro* colony formation assays is shown. Fifty thousand TFTS66 and TFC7 cells per dish were grown under the Dox concentrations indicated and treated with the indicated concentrations of 5-FU for 72 h. At Day 10, colonies were counted. Throughout the experiments, cells were maintained in media containing HygB and G418. Each experiment was triplicated. **C.** Survival curves of TFTS66 and TCF7 cells exposed to 5-FU. The survival fractions were calculated as a percentage of the untreated (*i*.*e*. 0 μM 5-FU) control, and the mean values are plotted against the 5-FU concentration: rectangle, TFTS66; circle, TFC7. The symbols are shaded according to the Dox concentrations. **D.** The IC50 value in each group was determined as the 5-FU concentration corresponding to 50% survival in the linearized survival curves. Standard error bars are shown at both ends of the linearized survival curves. **E.** The obtained IC50 values are plotted as a function of the TS expression level determined by immunoblotting (see Fig 2B): rectangle, TFTS66; circle, TFC7. The symbols are similarly shaded according to the Dox concentrations.

The sensitivity of TFTS66 cells to 5-FU was first examined using flowcytometry. It has been known that 5-FU treatment causes a marked accumulation of S-phase cells in cell populations sensitive to this agent [17]. We therefore treated TSTF66 and parental DLD-1 cells with different concentrations of 5-FU and analyzed them (Fig 4A). In DLD-1 cells, S phase cells accumulated according to the 5-FU concentrations, as expected. On the other hand, this phenomenon was not evident in TFTS66 cells, and the flowcytometric profiles were not largely different among the concentrations used, which indirectly suggests that TFTS66 cells are more resistant to 5-FU than DLD-1 cells. Next, we assessed the 5-FU sensitivity of TFTS66 cells by colony formation assays. TFTS66 and TCF7 cells, exposed to different concentrations of Dox, were treated with various concentrations of 5-FU for 72 h and plated (Fig 4B). Four days after the removal of 5-FU from the media, formed colonies were counted. Survival curves were plotted using mean values (Fig 4C). Each data point had a relatively small standard error (see Fig 4D). The survival of TFTS66 cells was apparently better than that of TFC7 cells, and, best in the Dox0 state. The survival of TFC7 cells did not differ, irrespective of the Dox concentration, which does imply that there were no synergistic effects between Dox and 5-FU. Remarkably, in the range above 0.1 ng/ml of Dox, the survival curves of TFTS66 cells were not largely different, although there was an evident difference between Dox0 and Dox0.05. Indeed, the change in TS expression is most dynamic between Dox0 and Dox0.05. However, the difference between Dox0.1 and Dox1 is also evident (see Fig 2B). Despite of the dynamic changes in TS expression, the survival of TFTS66 cells exposed to the high concentrations of Dox was invariable, which may be caused by the Dox-induced drug resistance suggested above. The effects of 5-FU might have been attenuated due to the induction of genes activating cellular transport, detoxification or those inhibiting cell death (see S1 Table). Therefore, we further examined the data of TFTS66 cells at Dox0, 0.05 and 0.1 and those of TFC7 cells. The IC50, the 5-FU concentration that corresponds to 50% survival, was determined from the linearized survival curves crossing 50% survival (Fig 4D), and plotted in a two-dimensional diagram of IC50 versus TS expression (Fig 4E). Intriguingly, the three data points, TFTS66 at Dox 0 and Dox0.05 and TFC7 at Dox0/0.1 (averaged), were on a straight line (R^2^ = 0.998). In other words, there was a highly linear relationship between TS expression and IC50 for 5-FU. The data point for TFTS66 cells at Dox0.1 was also plotted on the same diagram. However, because IC50 was at the same level between Dox0.1 and Dox0.05, this data point was not on the line, which again confirms that the survival of TFTS66 cells is invariable at the concentrations above 0.1ng/ml of Dox. It has thus been shown that in the TFTS66 transformant cellular sensitivity to 5-FU changes accordingly when TS expression level is modulated, although 5-FU sensitivity in TFTS66 cells expressing low levels of TS was not fully evaluated due to the limits of the system. These *in vitro* findings overall suggest that TS expression is a determinant of 5-FU sensitivity, at least in this particular genetic background.

### Responses of TFTS66 cell xenografts to 5-FU in nude mice

We further extended our study to an *in vivo* system using nude mice and cell xenografts. It is widely known that parental DLD-1 cells can readily form tumors in nude mice. We therefore inoculated TFTS66 cells subcutaneously into nude mice and found that TFTS66 cells similarly form tumor xenografts. First, we observed TS expression in TFTS66 cell xenografts using immunohistochemistry. TS proteins were expressed in cells of the xenografts at a very high level, reflecting the *in vitro* results (data not shown). Next, we examined the toxicity of Dox in nude mice and its effects on TS expression in tumor xenografts. The toxicity of this tetracycline antibiotic was not ignorable. However, its effects to suppress TS expression in TFTS66 cell xenografts were evident, and we observed that TS expression was suppressed to an extremely low level in the xenograft tissues in the animals administered with effective concentrations of Dox (data not shown). Finally, the Dox dose was fixed at 60 mg/kg per day. Unexpectedly, TS expression in xenografts under the Dox-free conditions tended to be gradually decreased as time passed (data not shown), which may be because HygB and G418 were not administered to the animals due to their toxicity. Therefore, the experiments were finished within two weeks.

**Fig 5:**
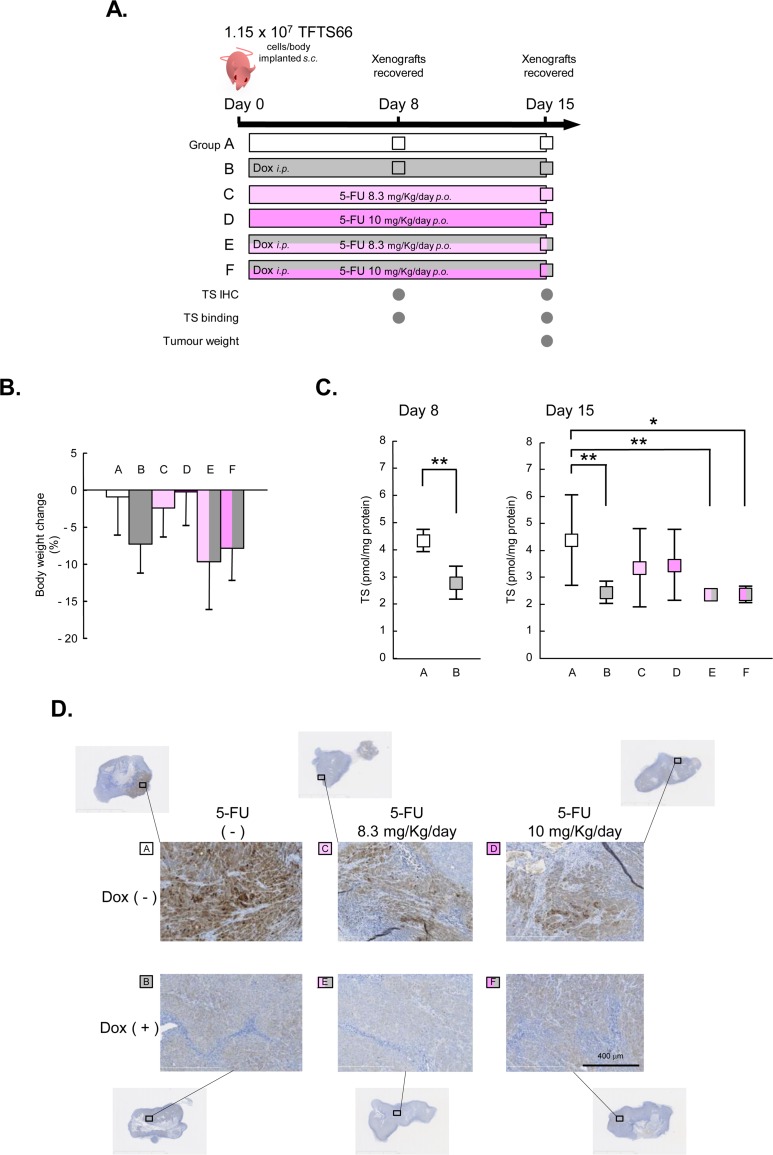
TFTS66 cell xenografts in nude mice. **A.** The design of the *in vivo* assays is shown. Seven-week old nude mice were randomly assigned to six groups. TFTS66 cells were subcutaneously inoculated in each animal. Dox and 5-FU were administered to the animals, intraperitoneally and orally, respectively. 5-FU doses are expressed as those of tegafur. TS binding assay and immunohistochemistry were done at day 4 (*n* = 4, group A and B) and day 8 (*n* = 8). At day 15, the weight of the tumor xenografts was measured. **B.** Body weight was measured twice a week, and the changes are shown as means with standard errors. **C.** The quantity of TS protein (TS_free_, see Materials and methods) in the xenograft tissues was determined by TS binding assay. Means with standard errors are shown, and significant differences are indicated with asterisks: *, p<0.05; **, p<0.01. **D.** Immunohistochemistry using antibody against TS. TS antigens were visualized as brown staining. Representative results are shown (scale bar: 400 μm). 5-FU doses are expressed as those of tegafur.

The antitumor effects of 5-FU were thus compared in TFTS66 cell xenografts that expressed TS at a very high level and those with an extremely low TS expression (Fig 5A). 5-FU was orally administered as a combined formulation of tegafur (an oral prodrug of 5-FU), CDHP and oxonate, which is now known as ‘S-1’ [9] and widely used for treatment of various human cancers. The two 5-FU doses (expressed as those of tegafur), 8.3 and 10.0 mg/kg/day, were chosen. Eight or twelve animals were assigned to each of the six groups. Although the experiments were well tolerated by most of the animals, weight loss was evident, particularly in those administered with Dox (Fig 5B), and, in those administered with Dox and 5-FU 10.0 mg/kg/day, three animals succumbed (group F, Table 1). TS expression, assessed by TS binding assay (Fig 5C), was well under control throughout the experiments. TS expression was suppressed to a very low level in the animals administered with Dox, whereas being maintained at relatively high levels in those without the Dox treatment, although the variance was not small in the latter groups at Day 15 (Fig 5C). The results of immunohistochemistry confirmed these findings, and intratumoral heterogeneity was observed (Fig 5D). Finally, the tumor weight in the animals of each group was measured and compared (Table 1). Importantly, Dox itself exhibited no significant growth inhibitory effects on tumor xenografts (compare Group A and B in Table 1), which is highly parallel to our *in vitro* data (compare Dox0 and 0.1/TFC7 in Fig 4). The effects of 5-FU was evident in Group E comprising animals administered with Dox and 5-FU 8.3 mg/kg/day. A significant inhibition of tumor growth was observed in the animals of this group, compared to those administered with only 8.3 mg/kg/day of 5-FU (Group C) (p = 0.008). Tumor growth was markedly inhibited also in Group F, which was, however, statistically not significant (p = 0.28), presumably due to the limited number of animals. Thus, using TSTF66 cell xenografts, we confirmed that cells expressing TS at a high level are more resistant to 5-FU than those with low TS expression also *in vivo*. These *in vitro* and *in vivo* observations are highly parallel and clearly suggest that TS expression is a determinant of 5-FU sensitivity in cells, at least in this specific genetic background.

## Discussion

Numerous clinical studies have been done on TS expression in human cancers, in order to clarify its significance as a determinant of 5-FU sensitivity of tumors. However, the data in the literature does not appear consistent (S2 Table). Since there is a variability in the genetic backgrounds of the subject populations and, in addition, of tumors, inductive clinical approaches clearly have limits. For the purpose of clarifying the relationship between expression of a single gene and its effects, transgene-based approaches are appropriate, and switchable transgenes such as those in Tet-ON/OFF systems [18–20], which can switch gene expression in a single genetic background, are more advantageous. TS transgene had not been addressed for long, but Johnston and colleagues [8] first reported their Tet-OFF system driving a TS transgene in 2001. This pioneer study provided a basis for our understanding of the significance of TS. However, several methodological improvements were possible in their study. In particular, we point out that two quantitatively unknown levels of gene expression were compared, and that it is therefore unclear how widely TS expression was modulated. In order to improve this problem, quantitative assessment of gene expression is prerequisite, and we used quantitative immunoblotting in this study. Our assays have elucidated that TS expression in TFTS66 cells was widely modulated, and that the dynamic range covered approximately 15-fold of the basal expression level (Fig 2B). This dynamic modulation of TS expression in TFTS66 cells was confirmed using several other quantitative assays. The quantitative assessment of expressed gene products also elucidated that gene expression can be widely and continuously modulated in the Tet systems. However, in fact, Tet-ON/OFF systems have thus far been used only for two-point comparisons. In this viewpoint, we first demonstrated that transgene expression is modulated in the ‘hyperbola-like’ dynamics in the Tet-OFF system (Fig 2B). The wide dynamic range of modulation allowed multiple observations and, consequently, lead to a multistep assessment of drug sensitivity in this study, which finally provided a quantitative relationship between TS expression and the 5-FU sensitivity. On the other hand, the above mentioned study assessed the 5-FU sensitivity of cells in a single comparison between two expression levels.

Needless to say, shortcomings are present also in our study. The Tet systems utilize tetracycline antibiotics that exhibit significant toxicity in eukaryotic cells. Indeed, the gene expression profiles obtained in TFTS66 cells exposed to Dox suggest that the cells reacted to this toxic agent, and that a mode of drug-resistance was induced. The genes induced by Dox exposure include ones regulating cellular transport, such as solute carrier family genes, which are now regarded as playing an important role in drug resistance in mammalian cells [21]. Genes inhibiting cell death were also induced. In particular, apoptosis inhibitor 5/antiapoptosis clone-11 (Api5/Aac11) is known to suppress a specific mode of apoptosis in mammalian cells and thought to function in tumor development [22]. It has also been shown that Api5 is expressed in various human tumors including colorectal cancer [23]. Taking these into account, it appears probable that cells exposed to high concentrations of Dox are not comparable to those in low Dox concentrations. Indeed, the survival of TFTS66 cells did not change in the range above 0.1 ng/ml of Dox, whereas being markedly different between 0 and 0.05 ng/ml. The cellular mechanisms of drug resistance induced by Dox exposure may have been active also against 5-FU, and this may have caused the refractory sensitivity to 5-FU in TFTS66 cells. Thus, despite the widely modulated TS expression, its effects on the 5-FU sensitivity were not examined in the full dynamic range. But, the results obtained in the control transformant carrying an empty vector complemented the data, and we found that IC50 and TS expression are in a linear relationship. A similar shortcoming is identified in our animal model. As expected, Dox exhibited significant toxicity also in mice, and three animals indeed succumbed to the toxicity (see Table 1). Therefore, the range of available Dox concentrations was limited, and, consequently, a single working concentration was selected. Thus, TS expression was not modulated in a wide range in the animal model, as was done *in vitro*. In other words, the *in vivo* experiments were a two-point comparison. Another shortcoming is again related to drug toxicity. In order to maintain two different expression vectors, two different antibiotics need to be administered continuously to stable transformant cells in the Tet systems. However, these agents are also toxic and, therefore, were not administered to the mice carrying the xenografts, which may have caused a loss of the vectors and, consequently, lead to a gradual decrease in TS transgene expression in the xenografts. The observation time was therefore limited. However, the difference in TS expression was successfully maintained throughout the *in vivo* experiments, and the results were highly parallel to those obtained *in vitro*. One may be concerned that there might be some synergistic effects of Dox and 5-FU in the observed growth inhibition. However, this possibility has been excluded by our *in vitro* data. 5-FU sensitivity of the control TFC7 transformant was unchanged, irrespective of Dox administration (see Fig 4C, 4D and 4E). Thus, as expected, the Tet systems obviously have limits particularly when drug sensitivity is studied, although there is in fact no substitute at present.

Our *in vitro* data suggest that there is a linear relationship between TS expression and the 5-FU sensitivity in TFTS66 cells, which mathematically implies that the latter is a one-dimensional function of the former. However, this does not necessarily conclude that the 5-FU sensitivity is determined exclusively by TS expression. Needless to say, drug sensitivity of cells is determined by highly complicated mechanisms. Cellular sensitivity to a given agent may be a more complex multivariable function of various cellular factors. The linear relationship between TS expression and the 5-FU sensitivity in TFTS66 cells is, in this sense, an approximately one-dimensional, at least in the range of parameter values in our system. Nevertheless, this finding appears novel. The relationship between gene expression and cellular phenotypes has not long been approached quantitatively. This may be primarily because the biological phenomena have been described not quantitatively but qualitatively in many fields of biology. Gene expression is not exceptional and has been assayed semi-quantitatively in general. It is in fact not widely recognized that more quantitative immunoblotting is made possible by additional control experiments, *i*.*e*. titrations of cell lysates (see Fig 2A). We first addressed this problem using several quantitative assays of gene expression and elucidated a mathematical relationship between expression of a gene of interest and its related cellular phenotype. One important outcome of this approach is that the obtained relationship allows prediction of 5-FU sensitivity of a given tumor. This step is essential for the development of real predictive biomarkers. Various genetic events in tumor cells have been regarded as biomarker candidates for classical anticancer agents. However, none has been clinically established to date. TS expression is a typical example, as discussed above. *A priori* demonstration of its significance may again stimulate clinical research and application. However, for this, it is prerequisite to develop more quantitative assays of TS expression in clinically obtained samples. TS binding assay may be a candidate, but various validations are necessary. Before extending the research further, it is also necessary to test different ranges of TS expression in cells derived from different sources (*i*.*e*. different genetic backgrounds), in order to generalize and extrapolate the results of this study. These efforts may ultimately lead to the establishment of more relevant and reliable biomarkers and, consequently, to truly personalized approaches for more effective treatment of cancer patients.

## Supporting Information

S1 Table Genes up/downregulated by exposure to doxycycline in TFTS66 cells. (PDF)

S2 Table TS expression and 5-FU sensitivity in human cancers—a survey of the literature. (PDF)

## References

[1] Schiffer CA, Clifton IJ, Davisson VJ, Santi DV, Stroud RM (1995) Crystal structure of human thymidylate synthase: a structural mechanism for guiding substrates into the active site. Biochemistry 34: 16279–16287. 8845352 10.1021/bi00050a007

[2] Newman EM, Lu Y, Kashani-Sabet M, Kesavan V, Scanlon KJ (1988) Mechanisms of cross-resistance to methotrexate and 5-fluorouracil in an A2780 human ovarian carcinoma cell subline resistant to cisplatin. Biochem Pharmacol 37: 443–447. 3337743 10.1016/0006-2952(88)90212-2

[3] Yoshioka A, Tanaka S, Hiraoka O, Koyama Y, Hirota Y, Ayusawa D, et al (1987) Deoxyribonucleoside triphosphate imbalance. 5-Fluorodeoxyuridine-induced DNA double strand breaks in mouse FM3A cells and the mechanism of cell death. J Biol Chem 262: 8235–8241. 2954951

[4] Grem JL, Danenberg KD, Behan K, Parr A, Young L, Danenberg PV, et al (2001) Thymidine kinase, thymidylate synthase, and dihydropyrimidine dehydrogenase profiles of cell lines of the National Cancer Institute's Anticancer Drug Screen. Clin Cancer Res 7: 999–1009. 11309351

[5] Ciaparrone M, Quirino M, Schinzari G, Zannoni G, Corsi DC, Vecchio FM, et al (2006) Predictive role of thymidylate synthase, dihydropyrimidine dehydrogenase and thymidine phosphorylase expression in colorectal cancer patients receiving adjuvant 5-fluorouracil. Oncology 70: 366–377. 17179731 10.1159/000098110

[6] Bissoon-Haqqani S, Moyana T, Jonker D, Maroun JA, Birnboim HC (2006) Nuclear expression of thymidylate synthase in colorectal cancer cell lines and clinical samples. J Histochem Cytochem 54: 19–29. 15956025 10.1369/jhc.5A6642.2005

[7] Koopman M, Venderbosch S, Nagtegaal ID, van Krieken JH, Punt CJ (2009) A review on the use of molecular markers of cytotoxic therapy for colorectal cancer, what have we learned? Eur J Cancer 45: 1935–1949. doi: 10.1016/j.ejca.2009.04.023 19473832 10.1016/j.ejca.2009.04.023

[8] Longley DB, Ferguson PR, Boyer J, Latif T, Lynch M, Maxwell P, et al (2001) Characterization of a thymidylate synthase (TS)-inducible cell line: a model system for studying sensitivity to TS- and non-TS-targeted chemotherapies. Clin Cancer Res 7: 3533–3539. 11705873

[9] Shirasaka T, Shimamato Y, Ohshimo H, Yamaguchi M, Kato T, Yonekura K, et al (1996) Development of a novel form of an oral 5-fluorouracil derivative (S-1) directed to the potentiation of the tumor selective cytotoxicity of 5-fluorouracil by two biochemical modulators. Anticancer Drugs 7: 548–557. 8862723 10.1097/00001813-199607000-00010

[10] Ayusawa D, Takeishi K, Kaneda S, Shimizu K, Koyama H, Seno T (1984) Isolation of functional cDNA clones for human thymidylate synthase. J Biol Chem 259: 14361–14364. 6094554

[11] Kozak M (1987) Effects of intercistronic length on the efficiency of reinitiation by eucaryotic ribosomes. Mol Cell Biol 7: 3438–3445. 3683388 10.1128/mcb.7.10.3438-3445.1987 PMC367994

[12] Laemmli UK (1970) Cleavage of structural proteins during the assembly of the head of bacteriophage T4. Nature 227: 680–685. 5432063 10.1038/227680a0

[13] Okabe H, Koizumi K, Tsujimoto H, Fukushima M (2000) Epitope analysis and utility of monoclonal antibodies to native and recombinant human thymidylate synthase. Int J Mol Med 5: 133–138. 10639590 10.3892/ijmm.5.2.133

[14] Spears CP, Shahinian AH, Moran RG, Heidelberger C, Corbett TH (1982) In vivo kinetics of thymidylate synthetase inhibition of 5-fluorouracil-sensitive and -resistant murine colon adenocarcinomas. Cancer Res 42: 450–456. 6173112

[15] Bradford MM (1976) A rapid and sensitive method for the quantitation of microgram quantities of protein utilizing the principle of protein-dye binding. Anal Biochem 72: 248–254. 942051 10.1016/0003-2697(76)90527-3

[16] Dunlap RB, Harding NG, Huennekens FM (1971) Thymidylate synthetase from amethopterin-resistant Lactobacillus casei. Biochemistry 10: 88–97. 5538615 10.1021/bi00777a014

[17] Tokunaga E, Oda S, Fukushima M, Maehara Y, Sugimachi K (2000) Differential growth inhibition by 5-fluorouracil in human colorectal carcinoma cell lines. Eur J Cancer 36: 1998–2006. 11000583 10.1016/s0959-8049(00)00200-8

[18] Gossen M, Bujard H (1992) Tight control of gene expression in mammalian cells by tetracycline-responsive promoters. Proc Natl Acad Sci U S A 89: 5547–5551. 1319065 10.1073/pnas.89.12.5547 PMC49329

[19] Gossen M, Freundlieb S, Bender G, Muller G, Hillen W, Bujard H (1995) Transcriptional activation by tetracyclines in mammalian cells. Science 268: 1766–1769. 7792603 10.1126/science.7792603

[20] Burcin MM, O'Malley BW, Tsai SY (1998) A regulatory system for target gene expression. Front Biosci 3: c1–7. 9478886 10.2741/a258

[21] Sprowl JA, Mikkelsen TS, Giovinazzo H, Sparreboom A (2012) Contribution of tumoral and host solute carriers to clinical drug response. Drug Resist Updat 15: 5–20. doi: 10.1016/j.drup.2012.01.009 22459901 10.1016/j.drup.2012.01.009 PMC3348357

[22] Morris EJ, Michaud WA, Ji JY, Moon NS, Rocco JW, Dyson NJ (2006) Functional identification of Api5 as a suppressor of E2F-dependent apoptosis in vivo. PLoS Genet 2: e196 17112319 10.1371/journal.pgen.0020196 PMC1636698

[23] Koci L, Chlebova K, Hyzdalova M, Hofmanova J, Jira M, Kysela P, et al (2012) Apoptosis inhibitor 5 (API-5; AAC-11; FIF) is upregulated in human carcinomas in vivo. Oncol Lett 3: 913–916. 22741017 10.3892/ol.2012.593 PMC3362606

