# Supplementary material for: Dynamic Modulation of Thymidylate Synthase Gene Expression and Fluorouracil Sensitivity in Human Colorectal Cancer Cells
Source: PLoS One. 2015 Apr 16;10(4):e0123076. doi: 10.1371/journal.pone.0123076 (PMC4400010; doi:10.1371/journal.pone.0123076)
Supplement: S1 Table — (PDF) [file pone.0123076.s001.pdf]

S1 Table. Genes up/downregulated by exposure to doxycyclin in TFTS66 cells.

Gene title	Gene symbol	Chromosome	Gene function	Expression ratio	
				Dox 0.5 / 0	Dox 1.0 / 0
Upregulated					
Solute carrier family 35, member F5	SLC35F5	chr2q14.1	nucleotide-sugar transporter	4.04	3.97
Golgi autoantigen, golgin subfamily a, 8A	GOLGA8A	chr15q11.2	details unknown	3.98	4.16
Apoptosis inhibitor 5	API5	chr11p11.2	inferred from physical interaction	3.11	1.94
Golgi autoantigen, golgin subfamily a, 4	GOLGA4	chr3p22-p21.3	details unknown	2.73	2.49
Solute carrier family 7, (cationic amino acid transporter, y+ system) member 11	SLC7A11	chr16q22.1	amino acid transmembrane transporter activity	2.70	2.23
Fas apoptotic inhibitory molecule 3	FAIM3	chr1q32.1	details unknown	2.52	1.04
Solute carrier organic anion transporter family, member 1B3	SLCO1B3	chr12p12	organic anion transmembrane transporter activity	2.24	1.96
Downregulated					
Thymidylate synthetase	TYMS	chr18p11.32	methyltransferase activity	0.39	0.37
Folate receptor 1 (adult)	FOLR1	chr11q13.3-q14.1	folic acid binding	0.26	0.35
