# Supplementary material for: Dynamic Modulation of Thymidylate Synthase Gene Expression and Fluorouracil Sensitivity in Human Colorectal Cancer Cells
Source: PLoS One. 2015 Apr 16;10(4):e0123076. doi: 10.1371/journal.pone.0123076 (PMC4400010; doi:10.1371/journal.pone.0123076)
Supplement: S2 Table — (PDF) [file pone.0123076.s002.pdf]

**S2 Table. TS expression and 5-FU sensitivity in human cancers - a survey of the literature. [a]**

| Authors | Year | Tumour | Patient No. | Method | Response [b] | Outcome [c] (5-FU+) [d] | Outcome (5-FU-) [e] | Reference |
|---|---|---|---|---|---|---|---|---|
| Okonkwo A | 2001 | Rectal cancer | 25 | IHC | Yes* | - | - | 1 |
| Cascinu S | 2001 | CRC | 150 | IHC | - | Yes (DFS) | - | 2 |
| Edler D | 2002 | CRC | 862 | IHC | - | No (+DFS) | Yes | 3 |
| Aschele C | 2002 | CRC | 124 | IHC | Yes | Yes (+PFS) | - | 4 |
| Tajima Y | 2003 | GC | 137 | IHC | - | Yes* | - | 5 |
| Tahara M | 2004 | GC | 29 | IHC | No | - | - | 6 |
| Shintani Y | 2004 | NSCLC | 116 | IHC | - | - | Yes (DFS) | 7 |
| Kamoshida S | 2004 | CRC | 37 | IHC | Yes | - | - | 8 |
| Yu Z | 2005 | BC | 197 | IHC | - | Yes | - | 9 |
| Jakob C | 2005 | CRC | 65 | IHC | Yes | - | - | 10 |
| Kawano K | 2006 | SCC | 140 | IHC | - | Yes | - | 11 |
| Dotor E | 2006 | CRC | 129 | IHC | - | No (+DFS) | - | 12 |
| Fernandez-Contreras ME | 2006 | CRC | 140 | IHC | - | Yes (PFS) | - | 13 |
| Ajiki T | 2006 | Gall bladder cancer | 53 | IHC | - | Yes* | - | 14 |
| Sinicrope FA | 2006 | CRC | 320 | IHC | - | No (+DFS) | - | 15 |
| Harada K | 2006 | EC | 25 | IHC | - | Yes | - | 16 |
| Fukuda H | 2006 | GC | 62 | IHC | Yes | - | - | 17 |
| Nakano J | 2006 | NSCLC | 151 | IHC | - | Yes | - | 18 |
| Popat S | 2006 | CRC | 967 | IHC | - | No | - | 19 |
| Ciaparrone M | 2006 | CRC | 62 | IHC | - | Yes | - | 20 |
| Jensen SA | 2007 | CRC | 303 | IHC | - | Yes* | - | 21 |
| Kwon HC | 2007 | GC | 64 | IHC | No | No | - | 22 |
| Bertolini F | 2007 | CRC | 91 | IHC | No | No | - | 23 |
| Bendardaf R | 2007 | CRC | 39 | IHC | No | No (DFS) | - | 24 |
| Li Y | 2007 | PC | 52 | IHC | - | Yes (DFS) | - | 25 |
| Miyoshi T | 2007 | NSCLC | 54 | IHC | - | Yes | - | 26 |
| Toi M | 2007 | BC | 192 | IHC | - | No (DFS) | Yes (DFS) | 27 |
| Bendardaf R | 2007 | CRC | 73 | IHC | Yes | Yes (DFS) | - | 28 |
| Kim MK | 2008 | EC | 108 | IHC | Yes | No | - | 29 |
| Negri FV | 2008 | CRC | 57 | IHC | Yes | - | - | 30 |
| Lee J | 2008 | GC | 467 | IHC | - | No | - | 31 |
| Soong R | 2008 | CRC | 945 | IHC | - | No | Yes* | 32 |
| Zheng Z | 2008 | NSCLC | 160 | IHC | - | Yes | - | 33 |
| Ceppi P | 2008 | GEP & lung NET | 116 | IHC | - | Yes (+PFS) | - | 34 |
| Honda J | 2008 | BC | 25 | IHC | No | - | - | 35 |
| Kuo SJ | 2008 | BC | 129 | IHC | - | Yes | - | 36 |
| Gosens MJ | 2008 | CRC | 38 | IHC | - | No | - | 37 |
| Bendardaf R | 2008 | CRC | 67 | IHC | - | No/Yes (DFS) | - | 38 |
| Li Y | 2009 | Bladder cancer | 67 | IHC | - | Yes (DFS) | - | 39 |
| Moreira LR | 2009 | CRC | 32 | IHC | - | No | - | 40 |
| Kim SH | 2009 | CRC | 70 | IHC | Yes | Yes | - | 41 |
| Kim JS | 2009 | GC | 153 | IHC | - | No (+DFS) | - | 42 |
| Ren DN | 2009 | CRC | 150 | IHC | - | No | - | 43 |
| Kostopoulos I | 2009 | CRC | 498 | IHC | - | No (+DFS) | - | 44 |
| Cullen KJ | 2010 | Head and neck SCC | 262 | IHC | - | No (+DFS) | - | 45 |
| Lee HW | 2010 | NPC | 41 | IHC | - | No | - | 46 |
| Karlberg M | 2010 | CRC | 1389 | IHC | - | - | Yes | 47 |
| Lee S | 2010 | EC | 72 | IHC | Yes | Yes (PFS) | - | 48 |
| O'Toole D | 2010 | GEP NET | 18 | IHC | No | - | - | 49 |
| Choi IS | 2011 | GC | 41 | IHC | - | No (+PFS) | - | 50 |
| Jeong SH | 2011 | GC | 72 | IHC | - | No | - | 51 |
| Nishi M | 2011 | CCC | 29 | IHC | - | - | No (+DFS) | 52 |
| Yasumatsu R | 2011 | SCC | 47 | IHC | No | - | - | 53 |
| Lee SJ | 2011 | BC | 90 | IHC | - | Yes (PFS) | - | 54 |
| Conradi LC | 2011 | Rectal cacner | 208 | IHC | - | Yes (+DFS) | - | 55 |
| Kondo N | 2011 | PC | 106 | IHC | - | - | No | 56 |
| Kaira K | 2011 | CRC | 80 | IHC | - | - | Yes (PFS) | 57 |
| Noda E | 2012 | CRC | 39 | IHC | No | - | Yes (PFS) | 58 |
| van der Zee JA | 2012 | PC | 212 | IHC | - | Yes*/No*(+DFS) | Yes (PFS) | 59 |
| Formentini A | 2012 | CRC | 140 | IHC | - | Yes | Yes (PFS) | 60 |
| Yokota K | 2012 | Thymic carcinoma | 24 | IHC | - | No | Yes (PFS) | 61 |
| Arsenijevic T | 2012 | SCC | 41 | IHC | No | - | Yes (PFS) | 62 |
| Sulzyc-Bielicka V | 2013 | CRC | 189 | IHC | - | Yes (+DFS) | Yes (PFS) | 63 |

a Studies are limited to those that employed immunohistocemistry for TS expression analyses.
b Patient responses to chemotherapy including 5-FU: Yes, a positive correlation bewteen high TS expression and poor responses; No, a negative
c Patient outcomes defined as overall survival, unless otherwise indicated: DFS, disease-free survival; PFS, progreassion-free survival; Yes, a positive correlation bewteen high TS expression and poor patient outocomes; No, a negative correlation, Yes*, a positive correlation under specific conditions.
d The subjects were patients who underwent chemotherapy including 5-FU
e The subjects were patients who underwent surgery only.
f Other abbreviations: IHC, immunoshitochemistry; CRC, colorectal cancer; GC, gastric cancer; NSCLC, non-small cell lung cancer; BC, breast cancer; SCC, squamous cell carcinoma; EC, oesophageal cancer; GEP NET, gastroenteropancreatic neuroendocrine tumours; NPC, nasopharyngeal cancer; CCC, intrahepatic cholangiocell carcinoma; Pancreatic cancer, PC.

## References

1. Okonkwo A, Musunuri S, Talamonti M, Benson A, 3rd, Small W, Jr., et al. (2001) Molecular markers and prediction of response to chemoradiation in rectal cancer. Oncol Rep 8: 497-500.
2. Cascinu S, Graziano F, Valentini M, Catalano V, Giordani P, et al. (2001) Vascular endothelial growth factor expression, S-phase fraction and thymidylate synthase quantitation in node-positive colon cancer: relationships with tumor recurrence and resistance to adjuvant chemotherapy. Ann Oncol 12: 239-244.
3. Edler D, Glimelius B, Hallstrom M, Jakobsen A, Johnston PG, et al. (2002) Thymidylate synthase expression in colorectal cancer: a prognostic and predictive marker of benefit from adjuvant fluorouracil-based chemotherapy. J Clin Oncol 20: 1721-1728.
4. Aschele C, Debernardis D, Bandelloni R, Cascinu S, Catalano V, et al. (2002) Thymidylate synthase protein expression in colorectal cancer metastases predicts for clinical outcome to leucovorin-modulated bolus or infusional 5-fluorouracil but not methotrexate-modulated bolus 5-fluorouracil. Ann Oncol 13: 1882-1892.
5. Tajima Y, Shimoda T, Nakanishi Y, Yokoyama N, Tanaka T, et al. (2003) Association of gastric and intestinal phenotypic marker expression of gastric carcinomas with tumor thymidylate synthase expression and response to postoperative chemotherapy with 5-fluorouracil. J Cancer Res Clin Oncol 129: 683-690.
6. Tahara M, Ochiai A, Fujimoto J, Boku N, Yasui W, et al. (2004) Expression of thymidylate synthase, thymidine phosphorylase, dihydropyrimidine dehydrogenase, E2F-1, Bak, Bcl-X, and Bcl-2, and clinical outcomes for gastric cancer patients treated with bolus 5-fluorouracil. Oncol Rep 11: 9-15.
7. Shintani Y, Ohta M, Hirabayashi H, Tanaka H, Iuchi K, et al. (2004) Thymidylate synthase and dihydropyrimidine dehydrogenase mRNA levels in tumor tissues and the efficacy of 5-fluorouracil in patients with non-small-cell lung cancer. Lung Cancer 45: 189-196.
8. Kamoshida S, Matsuoka H, Shiogama K, Matsuyama A, Shimomura R, et al. (2004) Immunohistochemical analysis of thymidylate synthase, p16(INK4a), cyclin-dependent kinase 4 and cyclin D1 in colorectal cancers receiving preoperative chemotherapy: significance of p16(INK4a)-mediated cellular arrest as an indicator of chemosensitivity to 5-fluorouracil. Pathol Int 54: 564-575.
9. Yu Z, Sun J, Zhen J, Zhang Q, Yang Q (2005) Thymidylate synthase predicts for clinical outcome in invasive breast cancer. Histol Histopathol 20: 871-878.
10. Jakob C, Liersch T, Meyer W, Baretton GB, Hausler P, et al. (2005) Immunohistochemical analysis of thymidylate synthase, thymidine phosphorylase, and dihydropyrimidine dehydrogenase in rectal cancer (cUICC II/III): correlation with histopathologic tumor regression after 5-fluorouracil-based long-term neoadjuvant chemoradiotherapy. Am J Surg Pathol 29: 1304-1309.
11. Kawano K, Yanagisawa S, Kusukawa J, Sunagawa H, Shiba R, et al. (2006) Intratumoural expression of thymidylate synthase is an independent predictor of prognosis in patients with squamous cell carcinoma of the tongue: results from a retrospective study. Int J Oral Maxillofac Surg 35: 258-264.
12. Dotor E, Cuatrecases M, Martinez-Iniesta M, Navarro M, Vilardell F, et al. (2006) Tumor thymidylate synthase 1494del6 genotype as a prognostic factor in colorectal cancer patients receiving fluorouracil-based adjuvant treatment. J Clin Oncol 24: 1603-1611.
13. Fernandez-Contreras ME, Sanchez-Prudencio S, Sanchez-Hernandez JJ, Garcia de Paredes ML, Gisbert JP, et al. (2006) Thymidylate synthase expression pattern, expression level and single nucleotide polymorphism are predictors for disease-free survival in patients of colorectal cancer treated with 5-fluorouracil. Int J Oncol 28: 1303-1310.
14. Ajiki T, Hirata K, Okazaki T, Horiuchi H, Fujita T, et al. (2006) Thymidylate synthase and dihydropyrimidine dehydrogenase expressions in gallbladder cancer. Anticancer Res 26: 1391-1396.
15. Sinicrope FA, Rego RL, Halling KC, Foster NR, Sargent DJ, et al. (2006) Thymidylate synthase expression in colon carcinomas with microsatellite instability. Clin Cancer Res 12: 2738-2744.
16. Harada K, Kawashima Y, Yoshida H, Sato M (2006) Thymidylate synthase expression in oral squamous cell carcinoma predicts response to S-1. Oncol Rep 15: 1417-1423.
17. Fukuda H, Takiguchi N, Koda K, Oda K, Seike K, et al. (2006) Thymidylate synthase and dihydropyrimidine dehydrogenase are related to histological effects of 5-fluorouracil and cisplatin neoadjuvant chemotherapy for primary gastric cancer patients. Cancer Invest 24: 235-241.
18. Nakano J, Huang C, Liu D, Masuya D, Nakashima T, et al. (2006) Evaluations of biomarkers associated with 5-FU sensitivity for non-small-cell lung cancer patients postoperatively treated with UFT. Br J Cancer 95: 607-615.
19. Popat S, Chen Z, Zhao D, Pan H, Hearle N, et al. (2006) A prospective, blinded analysis of thymidylate synthase and p53 expression as prognostic markers in the adjuvant treatment of colorectal cancer. Ann Oncol 17: 1810-1817.
20. Ciaparrone M, Quirino M, Schinzari G, Zannoni G, Corsi DC, et al. (2006) Predictive role of thymidylate synthase, dihydropyrimidine dehydrogenase and thymidine phosphorylase expression in colorectal cancer patients receiving adjuvant 5-fluorouracil. Oncology 70: 366-377.
21. Jensen SA, Vainer B, Sorensen JB (2007) The prognostic significance of thymidylate synthase and dihydropyrimidine dehydrogenase in colorectal cancer of 303 patients adjuvantly treated with 5-fluorouracil. Int J Cancer 120: 694-701.
22. Kwon HC, Roh MS, Oh SY, Kim SH, Kim MC, et al. (2007) Prognostic value of expression of ERCC1, thymidylate synthase, and glutathione S-transferase P1 for 5-fluorouracil/oxaliplatin chemotherapy in advanced gastric cancer. Ann Oncol 18: 504-509.
23. Bertolini F, Bengala C, Losi L, Pagano M, Iachetta F, et al. (2007) Prognostic and predictive value of baseline and posttreatment molecular marker expression in locally advanced rectal cancer treated with neoadjuvant chemoradiotherapy. Int J Radiat Oncol Biol Phys 68: 1455-1461.
24. Bendardaf R, Elzagheid A, Lamlum H, Algars A, Korkeila E, et al. (2007) Thymidylate synthase expression in primary colorectal tumours is correlated with its expression in metastases. Scand J Gastroenterol 42: 471-476.
25. Li Y, Mizutani Y, Shiraishi T, Okihara K, Ukimura O, et al. (2007) Prognostic significance of thymidylate synthase expression in patients with prostate cancer undergoing radical prostatectomy. Urology 69: 988-995.
26. Miyoshi T, Kondo K, Toba H, Yoshida M, Fujino H, et al. (2007) Predictive value of thymidylate synthase and dihydropyrimidine dehydrogenase expression in tumor tissue, regarding the efficacy of postoperatively administered UFT (tegafur+uracil) in patients with non-small cell lung cancer. Anticancer Res 27: 2641-2648.
27. Toi M, Ikeda T, Akiyama F, Kurosumi M, Tsuda H, et al. (2007) Predictive implications of nucleoside metabolizing enzymes in premenopausal women with node-positive primary breast cancer who were randomly assigned to receive tamoxifen alone or tamoxifen plus tegafur-uracil as adjuvant therapy. Int J Oncol 31: 899-906.
28. Bendardaf R, Lamlum H, Ristamaki R, Korkeila E, Syrjanen K, et al. (2008) Thymidylate synthase and microsatellite instability in colorectal cancer: implications for disease free survival, treatment response and survival with metastases. Acta Oncol 47: 1046-1053.
29. Kim MK, Cho KJ, Kwon GY, Park SI, Kim YH, et al. (2008) ERCC1 predicting chemoradiation resistance and poor outcome in oesophageal cancer. Eur J Cancer 44: 54-60.
30. Negri FV, Campanini N, Camisa R, Pucci F, Bui S, et al. (2008) Biological predictive factors in rectal cancer treated with preoperative radiotherapy or radiochemotherapy. Br J Cancer 98: 143-147.
31. Lee J, Park CK, Park JO, Lim T, Park YS, et al. (2008) Impact of E2F-1 expression on clinical outcome of gastric adenocarcinoma patients with adjuvant chemoradiation therapy. Clin Cancer Res 14: 82-88.
32. Soong R, Shah N, Salto-Tellez M, Tai BC, Soo RA, et al. (2008) Prognostic significance of thymidylate synthase, dihydropyrimidine dehydrogenase and thymidine phosphorylase protein expression in colorectal cancer patients treated with or without 5-fluorouracil-based chemotherapy. Ann Oncol 19: 915-919.
33. Zheng Z, Li X, Schell MJ, Chen T, Boulware D, et al. (2008) Thymidylate synthase in situ protein expression and survival in stage I nonsmall-cell lung cancer. Cancer 112: 2765-2773.
34. Ceppi P, Volante M, Ferrero A, Righi L, Rapa I, et al. (2008) Thymidylate synthase expression in gastroenteropancreatic and pulmonary neuroendocrine tumors. Clin Cancer Res 14: 1059-1064.
35. Honda J, Sasa M, Moriya T, Bando Y, Hirose T, et al. (2008) Thymidine phosphorylase and dihydropyrimidine dehydrogenase are predictive factors of therapeutic efficacy of capecitabine monotherapy for breast cancer-preliminary results. J Med Invest 55: 54-60.
36. Kuo SJ, Wang HC, Chow KC, Chiou SH, Chiang SF, et al. (2008) Expression of rTSbeta as a 5-fluorouracil resistance marker in patients with primary breast cancer. Oncol Rep 19: 881-888.
37. Gosens MJ, Moerland E, Lemmens VP, Rutten HT, Tan-Go I, et al. (2008) Thymidylate synthase genotyping is more predictive for therapy response than immunohistochemistry in patients with colon cancer. Int J Cancer.
38. Bendardaf R, Ristamaki R, Syrjanen K, Pyrhonen S (2008) Bcl-2 expression significantly correlates with thymidylate synthase expression in colorectal cancer patients. World J Gastroenterol 14: 6218-6223.
39. Li Y, Li X, Dai H, Sun X, Li J, et al. (2009) Thymidylate synthase was associated with patient prognosis and the response to adjuvant therapy in bladder cancer. BJU Int 103: 547-552.
40. Moreira LR, Schenka AA, Latuff Filho P, Nascimento H, Passos Lima CS, et al. (2009) Correlation between thymidylate synthase protein expression and gene polymorphism with clinicopathological parameters in colorectal carcinoma. Int J Surg Pathol 17: 181-186.
41. Kim SH, Kwon HC, Oh SY, Lee DM, Lee S, et al. (2009) Prognostic value of ERCC1, thymidylate synthase, and glutathione S-transferase pi for 5-FU/oxaliplatin chemotherapy in advanced colorectal cancer. Am J Clin Oncol 32: 38-43.
42. Kim JS, Kim MA, Kim TM, Lee SH, Kim DW, et al. (2009) Biomarker analysis in stage III-IV (M0) gastric cancer patients who received curative surgery followed by adjuvant 5-fluorouracil and cisplatin chemotherapy: epidermal growth factor receptor (EGFR) associated with favourable survival. Br J Cancer 100: 732-738.
43. Ren DN, Kim IY, Koh SB, Chang SJ, Eom M, et al. (2009) Comparative analysis of thymidylate synthase at the protein, mRNA, and DNA levels as prognostic markers in colorectal adenocarcinoma. J Surg Oncol 100: 546-552.
44. Kostopoulos I, Karavasilis V, Karina M, Bobos M, Xiros N, et al. (2009) Topoisomerase I but not thymidylate synthase is associated with improved outcome in patients with resected colorectal cancer treated with irinotecan containing adjuvant chemotherapy. BMC Cancer 9: 339.
45. Cullen KJ, Schumaker L, Nikitakis N, Goloubeva O, Tan M, et al. (2009) beta-Tubulin-II expression strongly predicts outcome in patients receiving induction chemotherapy for locally advanced squamous carcinoma of the head and neck: a companion analysis of the TAX 324 trial. J Clin Oncol 27: 6222-6228.
46. Lee HW, Hwang YH, Han JH, Choi JH, Kang SY, et al. (2010) High expression of excision repair cross-complementation group 1 protein predicts poor outcome in patients with nasopharyngeal cancer. Oral Oncol 46: 209-213.
47. Karlberg M, Ohrling K, Edler D, Hallstrom M, Ullen H, et al. (2010) Prognostic and predictive value of thymidylate synthase expression in primary colorectal cancer. Anticancer Res 30: 645-651.
48. Lee S, Park YH, Kim KH, Cho EY, Ahn YC, et al. (2010) Thymidine synthase, thymidine phosphorylase, and excision repair cross-complementation group 1 expression as predictive markers of capecitabine plus cisplatin chemotherapy as first-line treatment for patients with advanced oesophageal squamous cell carcinoma. Br J Cancer 103: 845-851.
49. O'Toole D, Couvelard A, Rebours V, Zappa M, Hentic O, et al. (2010) Molecular markers associated with response to chemotherapy in gastro-entero-pancreatic neuroendocrine tumors. Endocr Relat Cancer 17: 847-856.
50. Choi IS, Lee HS, Lee KW, Kim H, Kim KH, et al. (2011) Biomarker analysis in patients with advanced gastric cancer treated with S-1 plus cisplatin chemotherapy: orotate phosphoribosyltransferase expression is associated with treatment outcomes. Med Oncol 28: 991-998.
51. Jeong SH, Han JH, Kim JH, Ahn MS, Hwang YH, et al. (2011) Bax predicts outcome in gastric cancer patients treated with 5-fluorouracil, leucovorin, and oxaliplatin palliative chemotherapy. Dig Dis Sci 56: 131-138.
52. Nishi M, Shimada M, Utsunomiya T, Morine Y, Imura S, et al. (2011) Role of dihydropyrimidine dehydrogenase and thymidylate synthase expression in immunohistochemistry of intrahepatic cholangiocarcinoma. Hepatol Res 41: 64-70.
53. Yasumatsu R, Nakashima T, Yamauchi M, Wakasaki T, Masuda M, et al. (2011) Thymidylate synthase expression as a predictor of clinical response to 5-fluorouracil-based chemoradiotherapy in patients with maxillary sinus squamous cell carcinoma. Auris Nasus Larynx 38: 387-391.
54. Lee SJ, Choi YL, Park YH, Kim ST, Cho EY, et al. (2011) Thymidylate synthase and thymidine phosphorylase as predictive markers of capecitabine monotherapy in patients with anthracycline- and taxane-pretreated metastatic breast cancer. Cancer Chemother Pharmacol 68: 743-751.
55. Conradi LC, Bleckmann A, Schirmer M, Sprenger T, Jo P, et al. (2011) Thymidylate synthase as a prognostic biomarker for locally advanced rectal cancer after multimodal treatment. Ann Surg Oncol 18: 2442-2452.
56. Kondo N, Murakami Y, Uemura K, Sudo T, Hashimoto Y, et al. (2011) Prognostic impact of dihydropyrimidine dehydrogenase expression on pancreatic adenocarcinoma patients treated with S-1-based adjuvant chemotherapy after surgical resection. J Surg Oncol 104: 146-154.
57. Kaira K, Okumura T, Ohde Y, Takahashi T, Murakami H, et al. (2011) Prognostic significance of thymidylate synthase expression in the adjuvant chemotherapy after resection for pulmonary metastases from colorectal cancer. Anticancer Res 31: 2763-2771.
58. Noda E, Maeda K, Inoue T, Fukunaga S, Nagahara H, et al. (2012) Predictive value of expression of ERCC 1 and GST-p for 5-fluorouracil/oxaliplatin chemotherapy in advanced colorectal cancer. Hepatogastroenterology 59: 130-133.
59. van der Zee JA, van Eijck CH, Hop WC, van Dekken H, Dicheva BM, et al. (2012) Expression and prognostic significance of thymidylate synthase (TS) in pancreatic head and periampullary cancer. Eur J Surg Oncol 38: 1058-1064.
60. Formentini A, Henne-Bruns D, Kornmann M (2012) Thymidylate synthase and cyclin D1 protein expression in lymph node negative colorectal cancer: role as prognostic factors? Hepatogastroenterology 59: 1859-1864.
61. Yokota K, Sasaki H, Okuda K, Shitara M, Hikosaka Y, et al. (2012) Expression of thymidylate synthase and orotate phosphoribosyltransferase in thymic carcinoma. Exp Ther Med 4: 589-593.
62. Arsenijevic T, Micev M, Nikolic V, Gavrilovic D, Radulovic S, et al. (2012) Is there a correlation between molecular markers and response to neoadjuvant chemoradiotherapy in locally advanced squamous cell esophageal cancer? J BUON 17: 706-711.
63. Sulzyc-Bielicka V, Domagala P, Bielicki D, Safranow K, Domagala W (2013) Thymidylate synthase expression and p21(WAF1)/p53 phenotype of colon cancers identify patients who may benefit from 5-fluorouracil based therapy. Cell Oncol (Dordr) 37: 17-28.
